# The LXRB-SREBP1 network regulates lipogenic homeostasis by controlling the synthesis of polyunsaturated fatty acids in goat mammary epithelial cells

**DOI:** 10.1186/s40104-022-00774-4

**Published:** 2022-11-07

**Authors:** Wenying Zhang, Changhui Zhang, Jun Luo, Huifen Xu, Jianxin Liu, Juan J. Loor, Hengbo Shi

**Affiliations:** 1 Institute of Dairy Science, College of Animal Sciences, Zhejiang University, Hangzhou, 310058 China; 2 College of Animal Science and Technology, Northwest A&F University, Yangling, 712100 China; 3 College of Animal Science and Technology, Henan Agricultural University, Zhengzhou, 450046 China; 4 Key Laboratory of Molecular Animal Nutrition (Zhejiang University), Ministry of Education, Hangzhou, 310058, China; 5 Mammalian Nutrition Physiology Genomics, Department of Animal Sciences and Division of Nutritional Sciences, University of Illinois, Urbana, IL 61801 USA

**Keywords:** Elongase, Lipid homeostasis, Liver X receptor, Mammary gland, Polyunsaturated fatty acids

## Abstract

**Background:**

In rodents, research has revealed a role of liver X receptors (LXR) in controlling lipid homeostasis and regulating the synthesis of polyunsaturated fatty acids (PUFA). Recent data suggest that LXRB is the predominant LXR subtype in ruminant mammary cells, but its role in lipid metabolism is unknown. It was hypothesized that LXRB plays a role in lipid homeostasis via altering the synthesis of PUFA in the ruminant mammary gland. We used overexpression and knockdown of LXRB in goat primary mammary epithelial cells (GMEC) to evaluate abundance of lipogenic enzymes, fatty acid profiles, content of lipid stores and activity of the stearoyl-CoA desaturase (SCD1) promoter.

**Results:**

Overexpression of LXRB markedly upregulated the protein abundance of LXRB while incubation with siRNA targeting LXRB markedly decreased abundance of LXRB protein. Overexpression of LXRB plus T0901317 (T09, a ligand for LXR) dramatically upregulated *SCD1* and elongation of very long chain fatty acid-like fatty acid elongases 5–7 (*ELOVL 5–7*), which are related to PUFA synthesis. Compared with the control, cells overexpressing LXRB and stimulated with T09 had greater concentrations of C16:0, 16:1, 18:1n7,18:1n9 and C18:2 as well as desaturation and elongation indices of C16:0. Furthermore, LXRB-overexpressing cells incubated with T09 had greater levels of triacylglycerol and cholesterol. Knockdown of LXRB in cells incubated with T09 led to downregulation of genes encoding elongases and desaturases. Knockdown of LXRB attenuated the increase in triacylglycerol and cholesterol that was induced by T09. In cells treated with dimethylsulfoxide, knockdown of LXRB increased the concentration of C16:0 at the expense of C18:0, while a significant decrease in C18:2 was observed in cells incubated with both siLXRB and T09. The abundance of sterol regulatory element binding transcription factor 1 precursor (pSREBP1) and its mature fragment (nSREBP1) was upregulated by T09, but not LXRB overexpression. In the cells cultured with T09, knockdown of LXRB downregulated the abundance for pSREBP1 and nSREBP1. Luciferase reporter assays revealed that the activities of wild type *SCD1* promoter or fragment with SREBP1 response element (SRE) mutation were decreased markedly when LXRB was knocked down. Activity of the *SCD1* promoter that was induced by T09 was blocked when the SRE mutation was introduced.

**Conclusion:**

The current study provides evidence of a physiological link between the LXRB and SREBP1 in the ruminant mammary cell. An important role was revealed for the LXRB-SREBP1 network in the synthesis of PUFA via the regulation of genes encoding elongases and desaturases. Thus, targeting this network might elicit broad effects on lipid homeostasis in ruminant mammary gland.

**Supplementary Information:**

The online version contains supplementary material available at 10.1186/s40104-022-00774-4.

## Introduction

Lipids are one of the most-valuable components in dairy products, especially the polyunsaturated fatty acids (PUFA), which contribute to human health and animal lifespan [1, 2]. Although ruminant products contain low amounts of PUFA relative to non-ruminants, the fact that endogenous PUFA affect peroxisome proliferator activated receptor alpha and regulate hepatic lipid metabolism illustrates their potential role in cellular homeostasis of important organs [3, 4].

In non-ruminants, the activity of fatty acid (FA) desaturases and elongases is the rate-limiting step for the synthesis of PUFA [5]. The functional roles of FA desaturases and elongases, e.g., stearoyl-CoA desaturase 1 (SCD1), FA desaturase (FADS) and elongation of very long chain fatty acid-like fatty acid elongases (ELOVL) in ruminant mammary gland have been investigated [6–9]. It is evident that sterol regulatory element binding transcription factor 1 (SREBP1) and liver X receptor (LXR) play a crucial role in regulating the expression of *SCD1* [10, 11]. Although some evidence suggests that ELOVL promoters are targets of SREBP1 in rodents [12, 13] and goat [14], knowledge of how the synthesis of PUFA is regulated in the ruminant mammary gland is unclear.

The SREBP1 precursor (pSREBP1) is cleaved in a two-step process to become active, and is then translocated to the nucleus (nSREBP1) where it activates genes involved in biosynthesis of FA [15–17]. Data in rodents underscored an important role for SREBP1 in altering elongation of long chain fatty acids [18, 19]. For instance, in mouse [12] and goats [14], ELOVL6 is suggested to be a target of SREBP1 because its promoter contains SREBP1 response element (SRE). Besides SREBP1, at least in non-ruminants, the transcription factor liver X receptor (LXR) is also involved in lipogenesis in an SREBP-dependent and -independent manner [20]. Upon activation by oxysterols or synthetic agonists in humans and rodents, LXR binds to LXR response elements (LXRE) in the promoters of target genes as a heterodimeric complex with retinoid X receptor, leading to transcription of lipogenic enzymes [21]. Studies have identified LXRE in the SREBP1c promoter in goats [22], cows [23], and mice [24]. Along with the fact that the mouse ELOVL5 promoter is regulated directly by LXR-SREBP1c [25], these data indicated that LXR signaling might play an important role in regulating PUFA synthesis.

Two protein subtypes of LXR (LXRA and LXRB) have been identified. Evidence in humans and rodents supports a different functional role for these two subtypes [26, 27]. LXRA is required for the control of cholesterol metabolism in the liver [28] and mammary gland [29], while LXRB is a major regulator of fat storage in muscle and white adipose tissue [30]. Data from macrophages in LXRA^−/−^ and LXRB^−/−^ mice suggested an equivalent role for LXRA and LXRB on the regulation of lipogenic genes [31]. Recent data in goat mammary cells suggested a specific response of the SREBP1c promoter upon LXRB activation [32]. Although recent data in goat mammary cells confirmed the existence of SRE sites in the ELOVL6 promoter [14], the interaction between LXRB and SREBP1 on the alteration of PUFA profiles in ruminant mammary cells is unknown.

The main goal of the current study was to assess the transcriptional regulatory mechanisms in the LXRB-SREBP1c network that could alter PUFA composition in goat mammary epithelial cells (GMEC). To address this, we used in vitro culture of primary GMEC. The protein abundance of LXRB was altered through an adenoviral vector or small interference RNA in cells incubated with its pharmacological agonist (T0901317—T09). The data provided novel insights on the role of the LXRB-SREBP1c network in lipid homeostasis via controlling the synthesis of PUFA.

## Materials and methods

### Cell culture and treatment

The GMEC were isolated from lactating Saanen dairy goats as described previously [33]. Details of cell culture are described elsewhere [34]. Culture medium was composed of DMEM/F12 (Hyclone, Beijing, China), 10% fetal bovine serum (Hyclone, Beijing, China), hydrocortisone (5 mg/L, Sigma-Aldrich, St. Louis, MO, USA), insulin (5 mg/L, Sigma-Aldrich, St. Louis, MO, USA) and penicillin/streptomycin (10,000 unit/L, Harbin Pharmaceutical Group, Harbin, China). Lactogenic medium was composed of DMEM/F12, insulin (5 mg/L), hydrocortisone (5 mg/L), penicillin/streptomycin (10,000 units/L), prolactin (2 μg/mL, Sigma-Aldrich, St. Louis, MO, USA), and bovine serum albumin (1 g/L, Sigma-Aldrich, St. Louis, MO, USA). The GMEC were cultured in a lactogenic medium for 24 h before initial experiments to promote lactogenesis.

The LXRB was overexpressed with a recombinant adenoviral vector containing a FLAG tag epitope (Ad-LXRB), and was knockdown via RNA interference (siLXRB) as described previously [32]. For adenoviral infection, the GMEC cultured in six-well plates were incubated with adenovirus medium Ad-GFP or Ad-LXRB, respectively. For RNA interference, the GMEC cultured in six-well plates were transfected with siLXRB or control (siNC), respectively, using Lipofectamine® RNAiMAX according to the manufacturer’s protocol (Thermofisher, Waltham, MA, USA). Treated GMEC were cultured with 1 μmol/L T09 (Selleck, Shanghai, China; diluted in dimethyl sulfoxide, DMSO at final concentration 0.1%, Sigma-Aldrich, St. Louis, MO, USA) or control (DMSO at 0.1%) after 24 h of initial culture, and then harvested at 48 h (24 h later) for RNA extraction, lipid analysis and protein collection.

### RNA extraction and qPCR

Total RNA from GMEC was extracted using the RNA Prep pure cell Kit (Tiangen Biotech Co. Ltd., Beijing, China). Synthesis of cDNA from 1 μg RNA was conducted using the PrimeScript™ RT kit (Takara Bio Inc., Otsu, Japan) according to the manufacturer’s instructions. Quantitative real-time PCR (qPCR) was performed according to the manufacturer’s instructions using a kit (SYBR® Premix Ex Taq™ II, Perfect Real Time, Takara Bio Inc., Otsu, Japan).

Genes studied include those involved in elongation (*ELOVL5*, *ELOVL6*, *ELOVL7*), lipid droplet formation (*PLIN2*), FA desaturase (*SCD1*, *FADS1* and *FADS2*) triacylglycerol (*DGAT2*) and cholesterol synthesis (*ABCA1*). All qPCR reactions were performed in an ABI7500 (Thermofisher, Waltham, MA, USA) sequence detector. Ubiquitously expressed transcript was used as the internal control. Primer sequences are reported in Table S1 (Additional file 1). The specificity of each PCR primers was assessed through running 2% agarose gel and sequencing [35, 36]. The amplification efficiencies were assessed by serial dilution and are described in Table S1.

### Lipid analysis

Total cellular triacylglycerol (TAG) was extracted according to the GPO-Trinder triglyceride assay kit protocol (Applygen Technologies, Shanghai, China). The mass of TAG was determined according to the manufacturer's instructions on a micro-titer plate reader (BioTek Instruments, Inc., VT, USA). Quantification of total cellular TAG was normalized to protein concentration determined in each well using a BCA protein assay (Thermofisher, Waltham, MA, USA) according to the manufacturer's instructions.

Oil Red O staining was performed on GMEC according to a method described previously [37]. The images were captured using a Leica fluorescent microscope (DMI4000B, Weztlar, Germany).

For assay of FA profiles, collected GMEC were scraped off the culture dish using a 2-mL aliquot of 2.5% (vol/vol) vitriol:methanol. Then, total lipid extraction and methylation were performed according to Shi et al. [38]. Methylated lipid samples were analyzed using a Gas Chromatography-Mass Spectrometer (Agilent Technologies, Santa Clara, CA, USA) installed with an HP-88 column (100 m × 0.25 mm i.d. × 0.25 μm film thickness, Agilent Technologies) following a published procedure [8]. The relative proportion of each FA was determined as the ratio of the FA peak to the total peaks in each run. Data for each FA were analyzed as a proportion of the total FAs detected.

### Luciferase analysis

The fragments of wild type SCD1 promoter (SCD1-wild, containing site − 1713 to + 65) and the SRE site mutation (SCD1-SREM) were constructed into the pGL3-basic vector as described previously [10]. For the luciferase assay, the cells cultured in 48-well plates at 80%–90% confluence were co-transfected with 300 ng of the vector (SCD1-wild or SCD1-SREM) plus 10 ng of control vector (Renilla luciferase) per well using Lipofactamine 2000 reagent (Invitrogen, USA). Transfected GMEC were incubated with siRNA using Lipofectamine® RNAiMAX. The GMEC incubated with siRNA were treated with 1 μmol/L T09 (final concentration, diluted in DMSO) or control (DMSO) after 24 h of initial culture, and then harvested at 48 h. The dual-Luciferase Reporter assay kit (Thermofisher, Waltham, MA, USA) was used to measure luciferase activity on a Fluoroskan Ascent apparatus (Thermofisher, Waltham, MA, USA) according to the kit manufacturer’s protocol. The relative luciferase activity was calculated as the ratio of firefly luciferase compared with renilla luciferase activity.

### Western blotting

Total protein was extracted with radioimmunoprecipitation assay buffer (Solarbio Tech Co. Ltd., Beijing, China) supplemented with phenylmethanesulfonyl fluoride (Thermofisher, Waltham, MA, USA) at a final concentration of 1 mmol/L. The protein concentrations were measured using a BCA kit (Thermofisher, MA, USA) according to the manufacturer’s instructions, and 30 μg of total protein per lane was separated by 12% SDS-PAGE gels. The blots were incubated with antibodies against SREBP1 (ab3259, Abcam, Cambridge, UK; 1:500), LXRB (ER1912-56, Huabio, Hangzhou, China; 1:300), β-actin (CW0096, CW Biotech, Beijing, China; 1:1000) and FLAG (80010–1-RR, proteintech, Wuhan, China; 1:1000). The antibody against FLAG tag was used to measure the exogenous LXRB protein with FLAG tag. HRP-conjugated IgG secondary antibody was used (Proteintech, Wuhan, China 1:1000). Signals were detected using a chemiluminescent ECL system (Thermofisher, MA, USA) and visualized by autoradiography with a cold CCD camera (Bio-Rad, CA, USA).

### Statistical analysis

Treatments were replicated at least 3 times, and results are expressed as means ± standard error of the means (SEM). Data from qPCR were analyzed using the 2^−ΔΔCt^ method and normalized to the respective control (siNC + DMSO or Ad-GFP + DMSO) [39]. Densitometry values of western blots were measured using Image J software (version 2) [40]. Protein abundance was normalized to β-actin. All data are reported as means ± SEM. Statistical differences were determined with a one-way ANOVA (Tukey) using SPSS 19.0 (SPSS, Inc./IBM Corp., Chicago, IL, USA). Significance was declared at *P* < 0.05.

## Results

### LXRB activation increased expression of genes related to PUFA synthesis

**Fig. 1:**
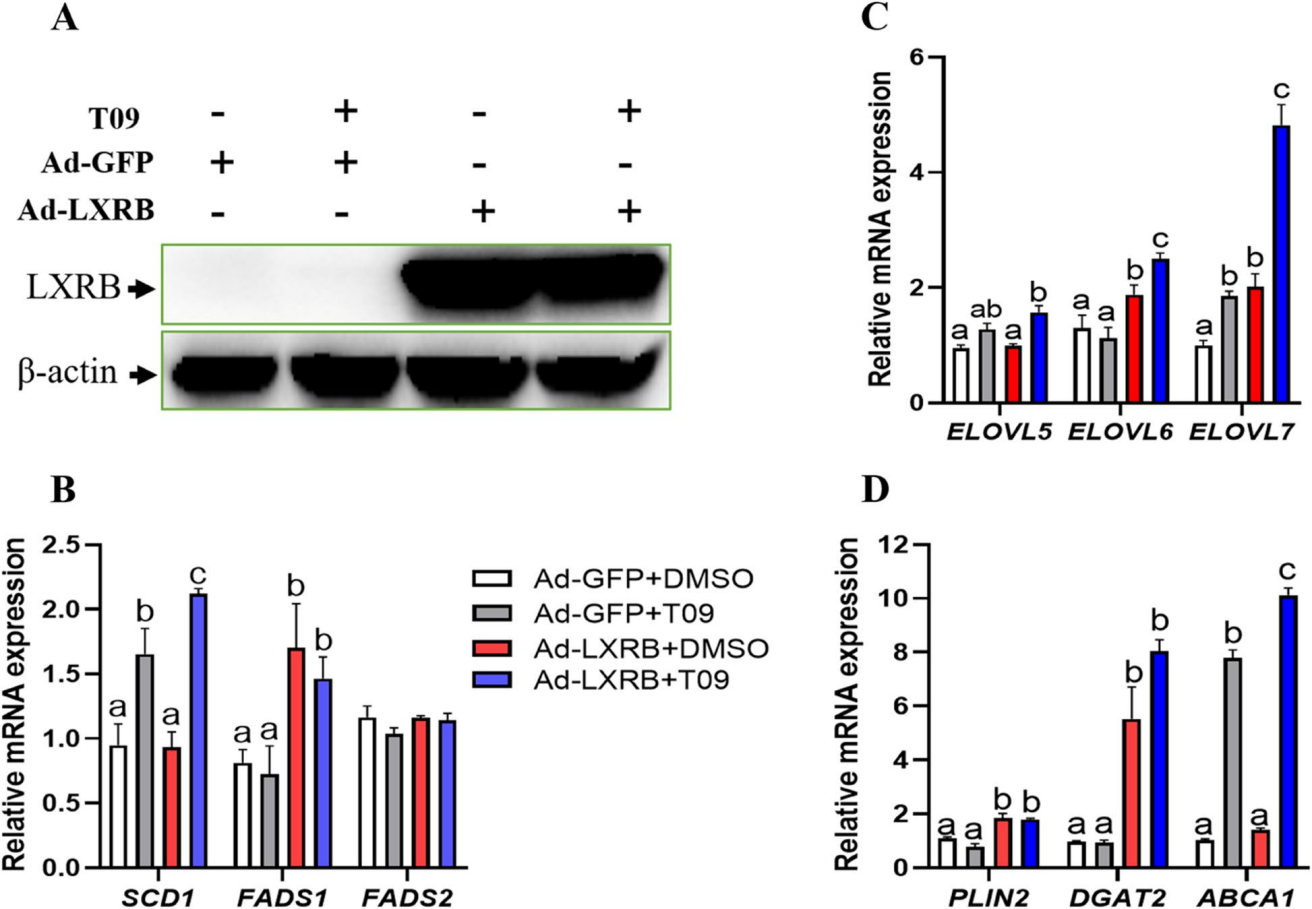
Changes in gene expression in goat mammary epithelial cells in response to overexpression of LXRB and T0901317 (T09). Panel **A**: Protein expression levels of LXRB; Panel **B**: mRNA expression of *SCD1*, *FADS1* and *FADS2*; Panel **C**: mRNA expression of *ELOVL5*, *ELOVL6* and *ELOVL7*. Panel **D**: mRNA expression of *PLIN2*, *DGAT2* and *ABCA1*. Treatments were replicated 3 times. Values are means ± SEM. The different letters denote significant (*P* < 0.05) differences and absence of letters denote absence of statistical differences

As shown in Fig. S1 (Additional file 1), *LXRB* mRNA was significantly upregulated (~ 50-fold) in the cells infected with Ad-LXRB (Ad-LXRB + DMSO) compared with the control (Ad-GFP + DMSO) while T09 alone had no effect compared with DMSO treatment (*n* = 3). Consistent with the mRNA level, the protein abundance of LXRB increased markedly when the GMEC were incubated with Ad-LXRB while T09 alone had barely an effect on LXRB protein compared with DMSO treatment (Fig. 1 A). Overexpression of LXRB in the absence of T09 had weak effects (*P* > 0.05) on *ELOVL5*, *SCD1, FADS2* and *ABCA1* abundance (Fig. 1 B, C and D). Addition of T09 to cultures overexpressing LXRB (Ad-LXRB + T09) upregulated the mRNA abundance of *ELOVL6* (*P* = 0.02), *ELOVL7* (*P* < 0.01), *SCD1* (*P* = 0.04), *FADS1* (*P* = 0.02), *PLIN2* (*P* = 0.02), *DGAT2* (*P* < 0.01) and *ABCA1* (*P* = 0.02), respectively, relative to the control (Ad-GFP + T09) (Fig. 1 B, C and D). Compared with control (Ad-GFP + DMSO), T09 treatment (Ad-GFP + T09) significantly enhanced the mRNA level of *ELOVL7* (*P* = 0.01), *SCD1*(*P* = 0.02) and *ABCA1* (*P* < 0.01) (Fig. 1 C and D) while LXRB overexpression alone increased mRNA expression of *ELOVL6* (*P* = 0.02), *ELOVL7* (*P* = 0.01), *FADS1*(*P* = 0.03), *PLIN2* (*P* = 0.01) and *DGAT2* (*P* < 0.01).

### LXRB enhanced cellular concentrations of TAG and cholesterol

**Fig. 2:**
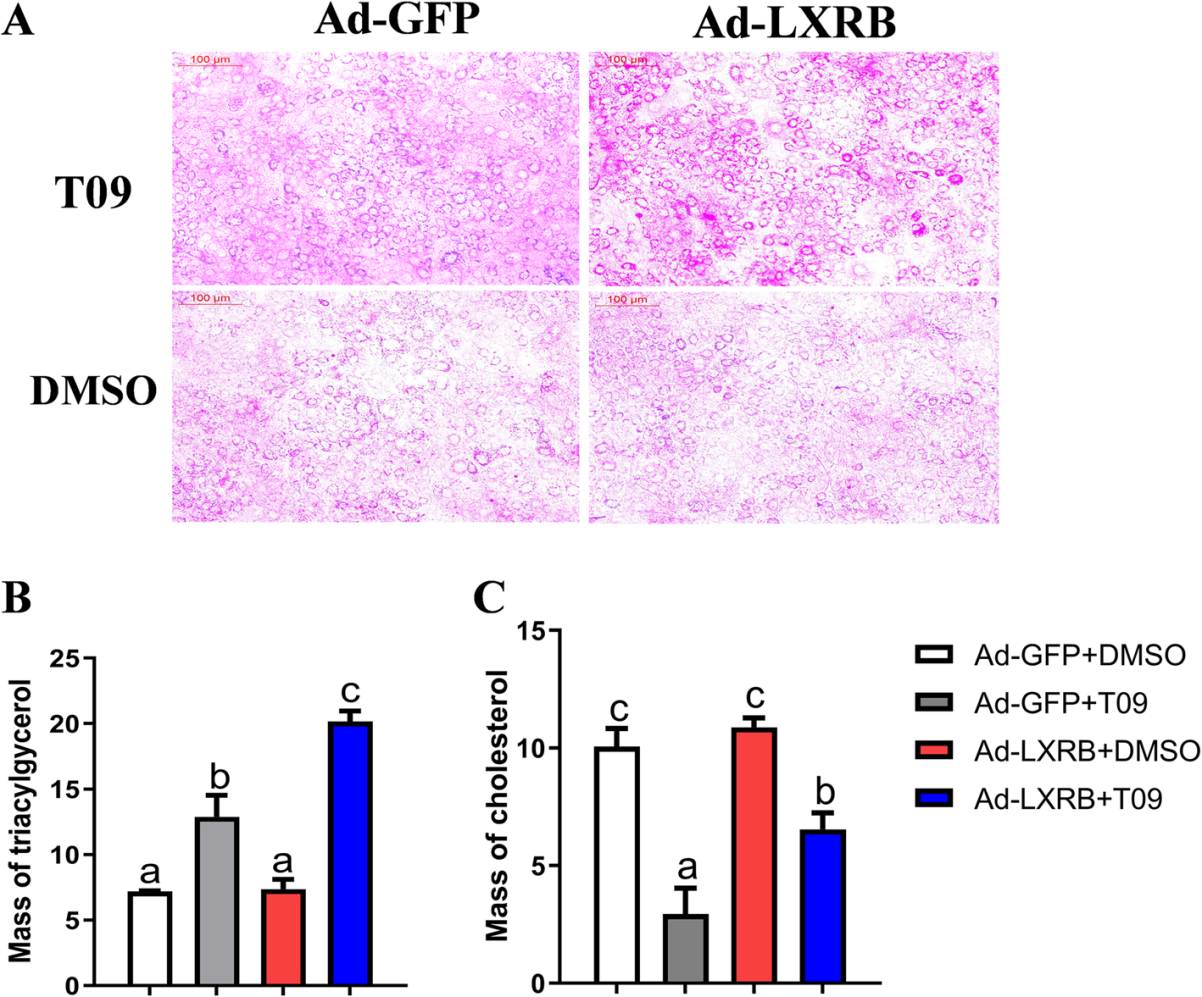
Overexpression of LXRB increases the content of triacylglycerol and cholesterol in goat mammary epithelial cells (GMEC). Panel **A**: lipid droplets in GMECs were detected by Oil Red O staining. Scale bar = 100 μm. Panel **B**: changes for the content of triacylglycerol in GMEC; Panel **C**: changes for the content of cholesterol in GMEC. Treatments were replicated 3 times. The Values are means ± SEM. The different letters denote significant (*P* < 0.05) differences and absence of letters denote absence of statistical differences

Oil Red O staining revealed that LXRB activation (with T09) enhanced cellular lipid accumulation in the presence or absence of Ad-LXRB (Fig. 2A, *n* = 3). Consistent with the data of Oil Red O staining, TAG analysis confirmed that cells incubated with T09 had higher TAG concentration (*P* = 0.02) compared with the control (Ad-GFP + DMSO) (Fig. 2 B). In addition, LXRB overexpression further enhanced the concentration of TAG in cells incubated with T09 (*P* = 0.01, Fig. 2 B). Overexpression of LXRB alone had no significant effect on concentration of TAG and cholesterol (Fig. 2 C). Compared with the control (Ad-GFP + DMSO), a lower level of cholesterol was observed in cells incubated with T09. In the cells incubated with T09, a significant increase in cholesterol was also observed (*P* = 0.02) when LXRB was overexpressed compared with control cells (Ad-GFP + T09).

### LXRB increased desaturation and elongation of FA

**Fig. 3:**
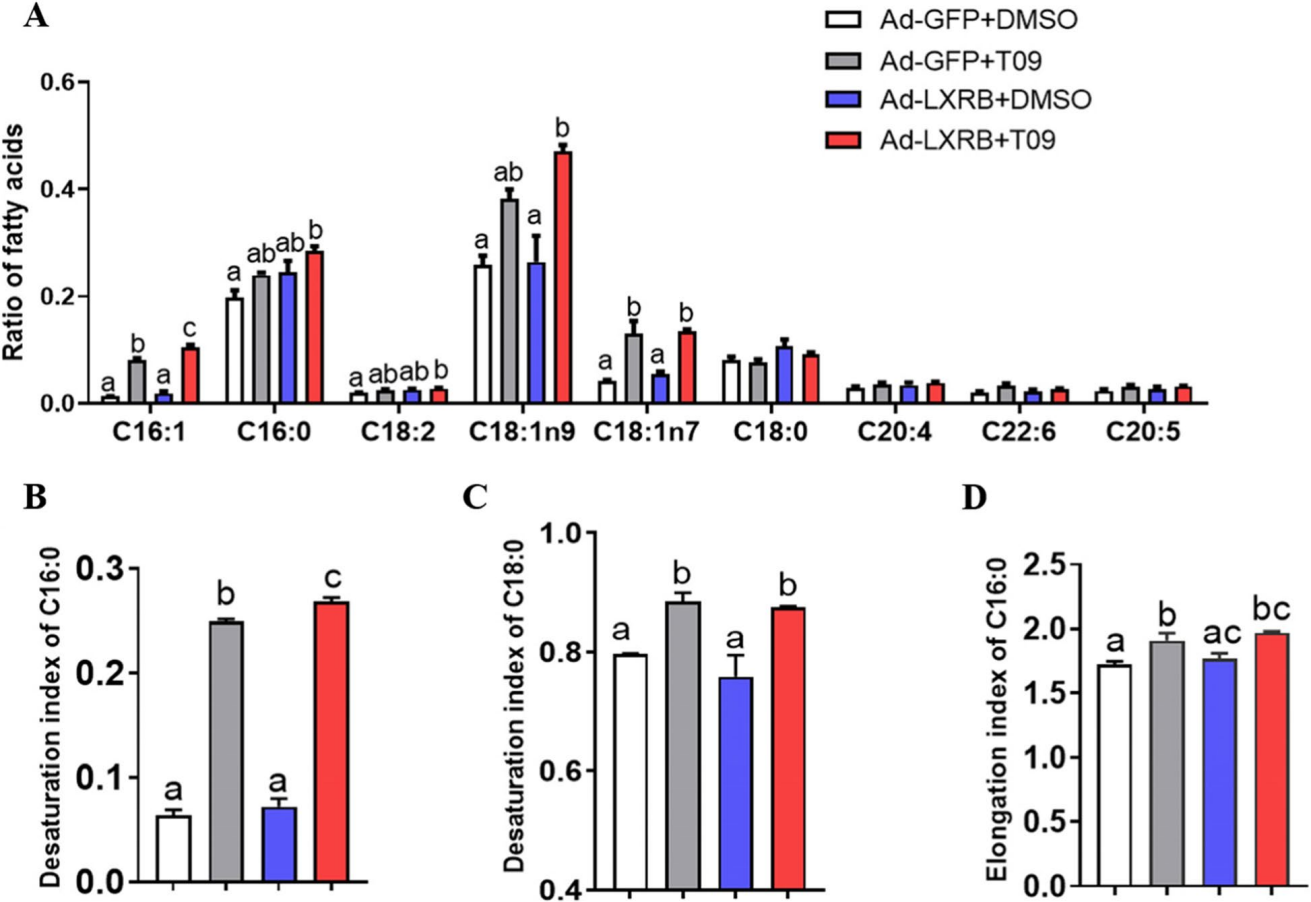
Overexpression of LXRB increases the content of fatty acids in goat mammary epithelial cells. Panel **A**: changes for the fatty acid profiles; Panel **B**: changes for the desaturation index of C16:0; Panel **C**: changes for the desaturation index of C18:0; Panel **D**: changes for the elongation index of C16:0. Treatments were replicated 5 times. The values are means ± SEM. The different letters denote significant (*P* < 0.05) differences and absence of letters denote absence of statistical differences

Compared with the control (Ad-GFP + DMSO group), incubation with T09 upregulated the ratio of C16:1 (*P* = 0.01) and C18:1n7 (*P* = 0.01) (Fig. 3A, *n* = 5) while overexpression of LXRB alone (Ad-LXRB + DMSO) had no effect (*P* > 0.05) on cellular FA profiles. Overexpressing LXRB in cells plus T09 increased significantly the ratio of C16:0 (*P* = 0.02), C16:1 (*P* < 0.01), C18:1n7 (*P* = 0.01), C18:1n9 (*P* = 0.01) and C18:2 (*P* = 0.03) compared with the control (Ad-GFP + DMSO). However, no significant changes (*P* > 0.05) for the concentrations of C18:0, C20:4, C22:6 and C20:5 were observed regardless of treatment with Ad-LXRB or T09. Consistent with the alteration of FA profiles, an increase in desaturation index of C16:0 (*P* < 0.01) and C18:0 (*P* = 0.04) was observed when treated with T09 (Fig. 3 B and C). The desaturation index of C16:0 was further enhanced by treatment with T09. In the cells overexpressing LXRB, T09 upregulated the desaturation index of C18:0 (*P* = 0.01), and had no significant effect on the elongation index of C16:0 (*P* > 0.05) (Fig. 3 D).

### Knockdown of LXRB decreased the expression of genes related to PUFA synthesis

**Fig. 4:**
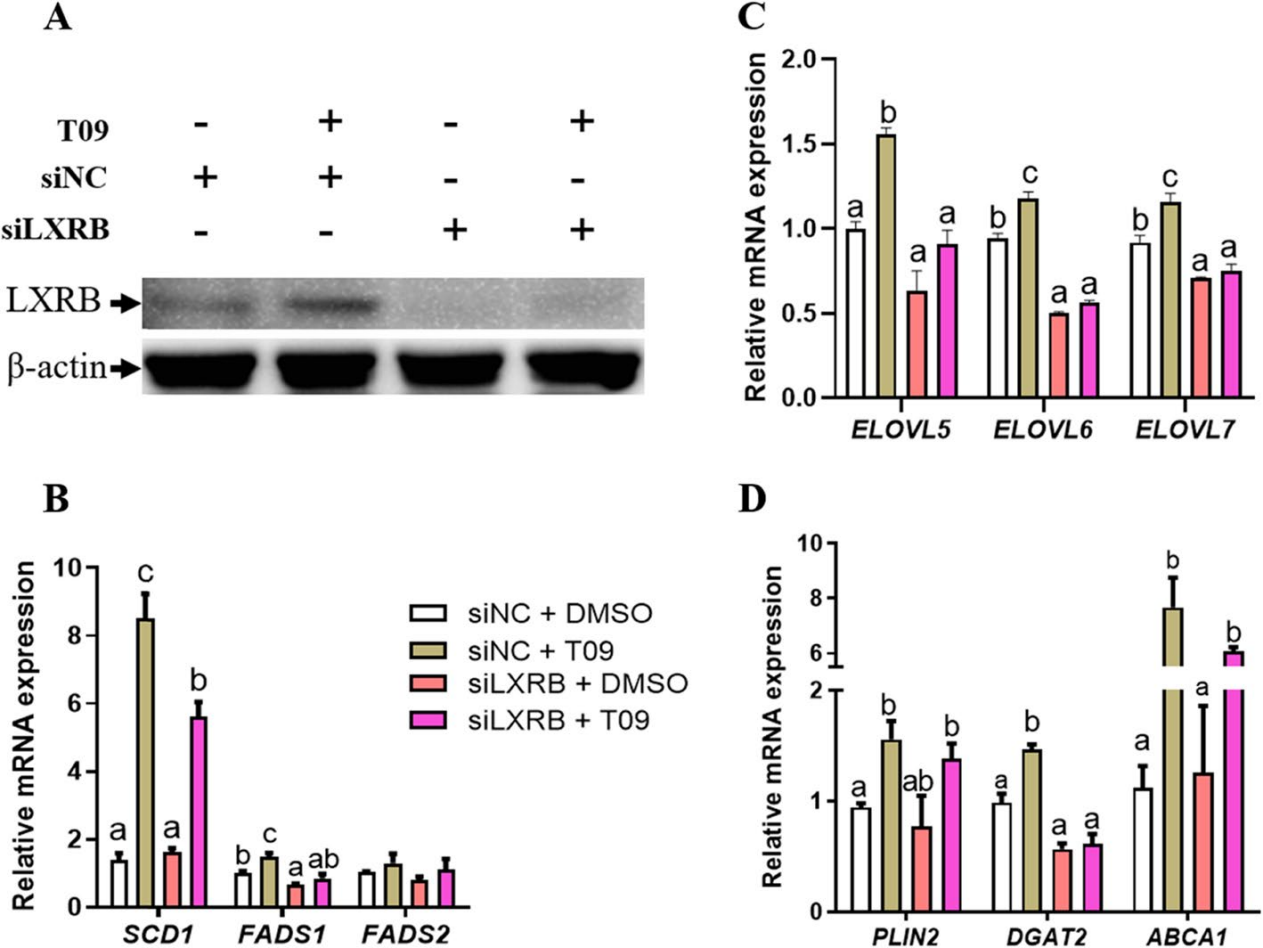
Changes in gene expression in goat mammary epithelial cells in response to knockdown of LXRB and T0901317 (T09). Panel **A**: Protein expression levels of LXRB; Panel **B**: mRNA expression of *SCD1*, *FADS1* and *FADS2*; Panel **C**: mRNA expression of *ELOVL5*, *ELOVL6* and *ELOVL7*. Panel **D**: mRNA expression of *PLIN2*, *DGAT2* and *ABCA1*. Treatments were replicated 3 times. Values are means ± SEM. The different letters denote significant (*P* < 0.05) differences and absence of letters denote absence of statistical differences

Consistent with the marked decrease in mRNA abundance of *LXRB* (Fig. S2, Additional file 1), protein abundance of LXRB was significantly increased when incubated with T09 and was markedly reduced by the siLXRB treatment (Fig. 4A, *n* = 3). Compared with the control (siNC + DMSO), LXRB knockdown (siLXRB + DMSO) did not significantly alter the mRNA abundance of *ELOVL5*, *SCD1*, *FADS2*, *PLIN2*, *DGAT2* and *ABCA1.* However, it significantly decreased *ELOVL6* (*P* = 0.02), *ELOVL7* (*P* = 0.04) and *FADS1* (*P* = 0.02) (Fig. 4 B, C and D)*.* In the cells incubated with T09, knockdown of LXRB significantly decreased the abundance of *ELOVL5* (*P* = 0.04), *ELOVL6* (*P* = 0.02), *ELOVL7* (*P* = 0.04), *SCD1* (*P* = 0.03), *FADS1* (*P* = 0.03) and *DGAT2* (*P* = 0.04) (Fig. 4 B, C and D)*.*

### Knockdown of LXRB altered the concentrations of TAG and cholesterol

**Fig. 5:**
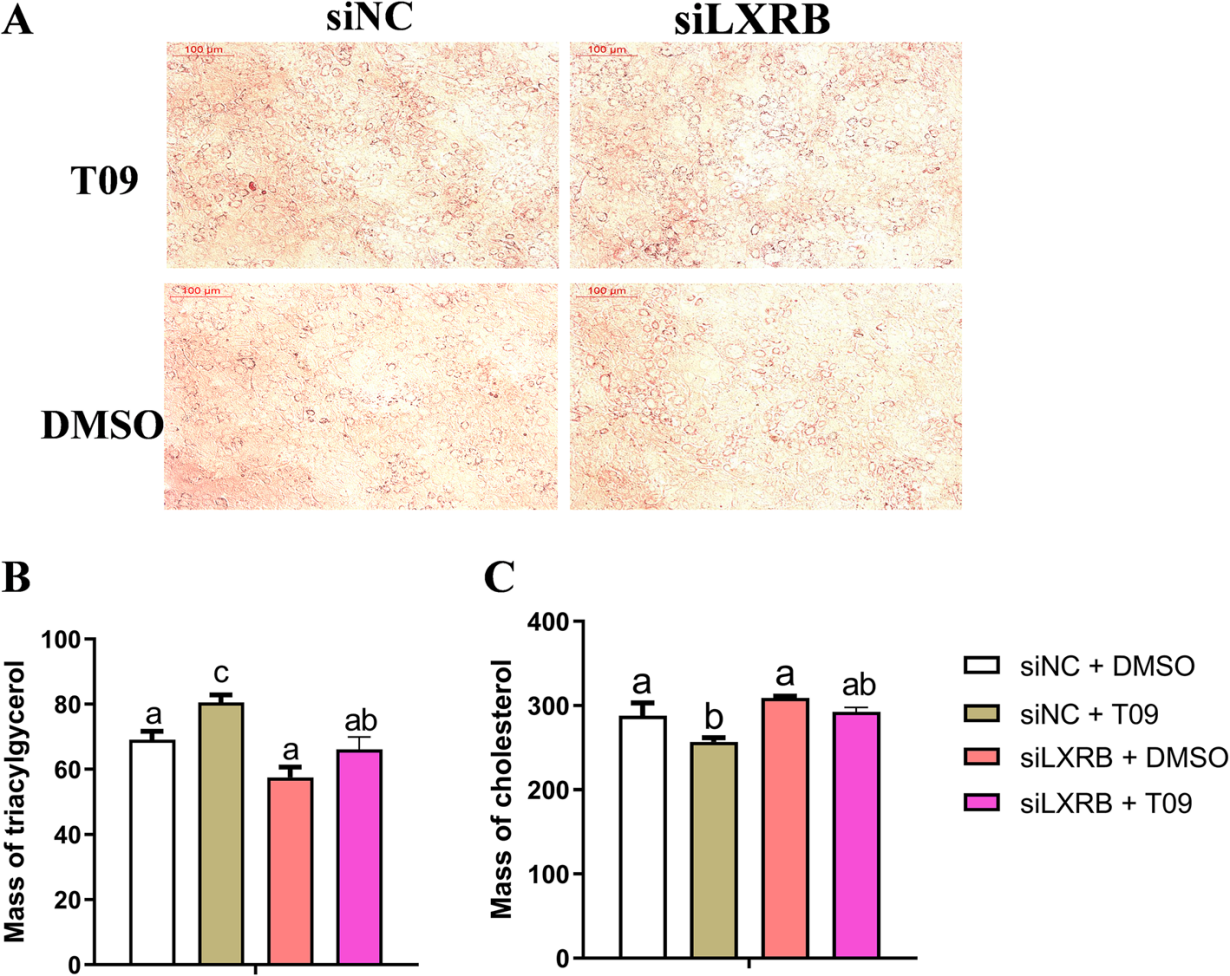
Knockdown of LXRB alters the content of triacylglycerol and cholesterol. Panel **A**: Lipid droplets in GMECs were detected by Oil Red O staining. Scale bar = 100 μm. Panel **B**: changes for the content of triacylglycerol; Panel **C**: changes for the content of cholesterol. Treatments were replicated 3 times. The Values are means ± SEM. The different letters denote significant (*P* < 0.05) differences and absence of letters denote absence of statistical differences

As shown with the Oil Red O staining and the TAG assay, knockdown of LXRB alone had no significant effect on lipid accumulation compared with the control group (Ad-GFP + DMSO) (Fig. 5A, B and C, *n* = 3). Downregulation of LXRB attenuated the stimulatory effect of T09 on TAG accumulation (*P* = 0.04, Fig. 5A and B). Compared with the control (siNC + DMSO), the addition of T09 led to a lower level of cholesterol (*P* = 0.03), while this effect was blocked in cells incubated with siLXRB (*P* = 0.07).

### Knockdown of LXRB altered the FA profiles

**Fig. 6:**
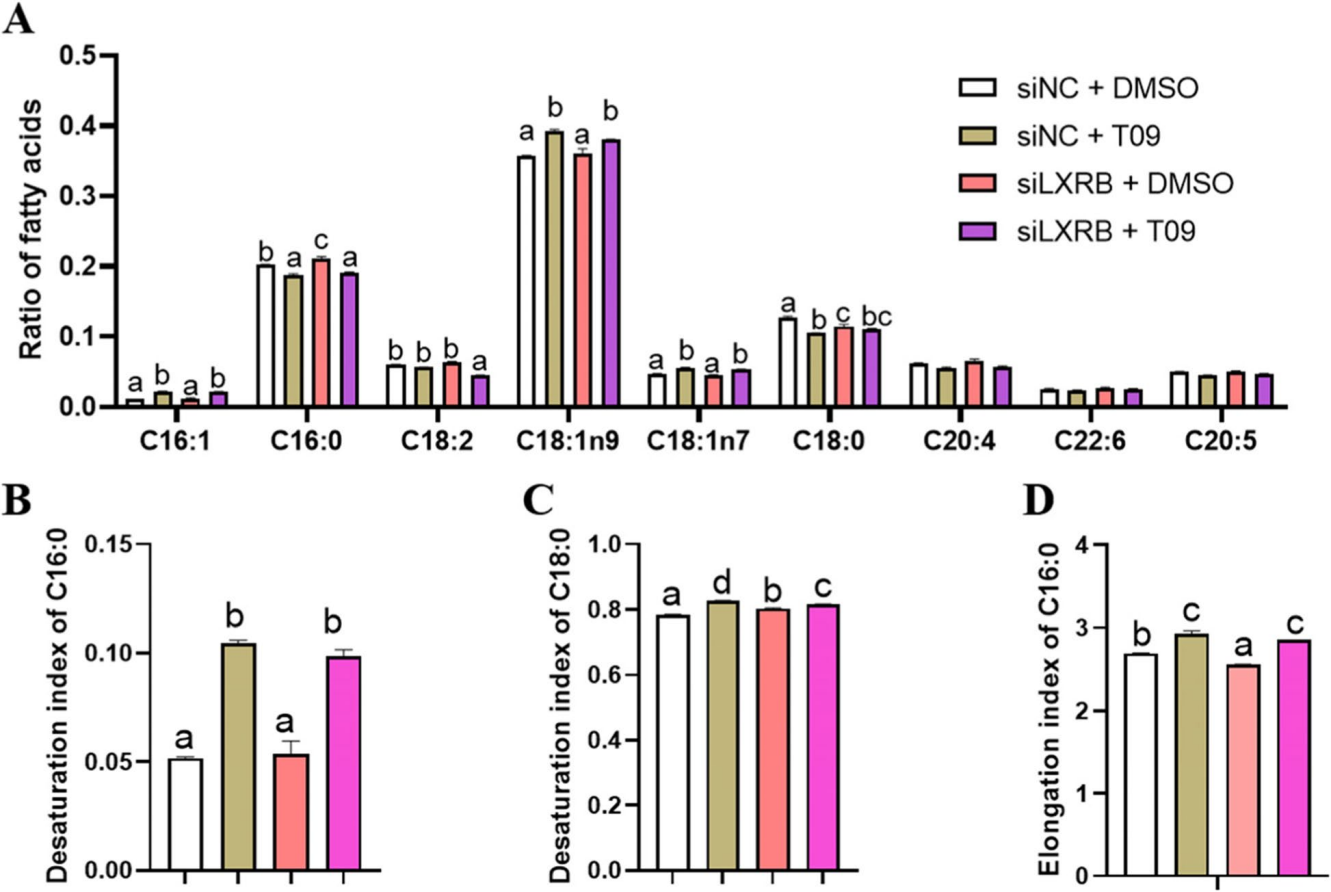
Knockdown of LXRB alters the fatty acid profiles. Panel **A**: changes for the fatty acid profiles; Panel **B**: changes for the desaturation index of C16:0; Panel **C**: changes for the desaturation index of C18:0; Panel **D**: changes for the elongation index of C16:0. Treatments were replicated 5 times. The Values are means ± SEM. The different letters denote significant (*P* < 0.05) differences and absence of letters denote absence of statistical differences

Compared with control (siNC + DMSO), knockdown of LXRB alone had no significant effect on the ratio of C16:1, C18:2. C18:1n9, C18:1n7, C20:4, C22:6 and C20:5 (Fig. 6A, *n* = 5). An increase in C16:0 (*P* = 0.03) at the expense of C18:0 (*P* = 0.04) was observed in the cells incubated with siLXRB alone. In the cells containing T09 and incubated with siLXRB the ratio of C18:2 (*P* = 0.04) decreased, but there was no effect on the other FA (siLXRB + T09 group vs siNC + T09 group). Incubation with siLXRB alone had no effect on desaturation of C16:0 (*P* = 0.98) while an increase in desaturation of C18:0 (*P* < 0.001) was observed compared with the control (siNC + DMSO) (Fig. 6 B and C). In the cells containing T09, siLXRB incubation had no significant effect on desaturation of C16:0 (*P* = 0.64), but decreased desaturation of C18:0 (*P* < 0.01). A decrease in elongation of C16:0 was observed in the cells incubated with siLXRB alone (*P* < 0.01), and was blocked by the addition of T09 (Fig. 6 D).

### Full LXRB activation is dependent on SREBP1 protein

**Fig. 7:**
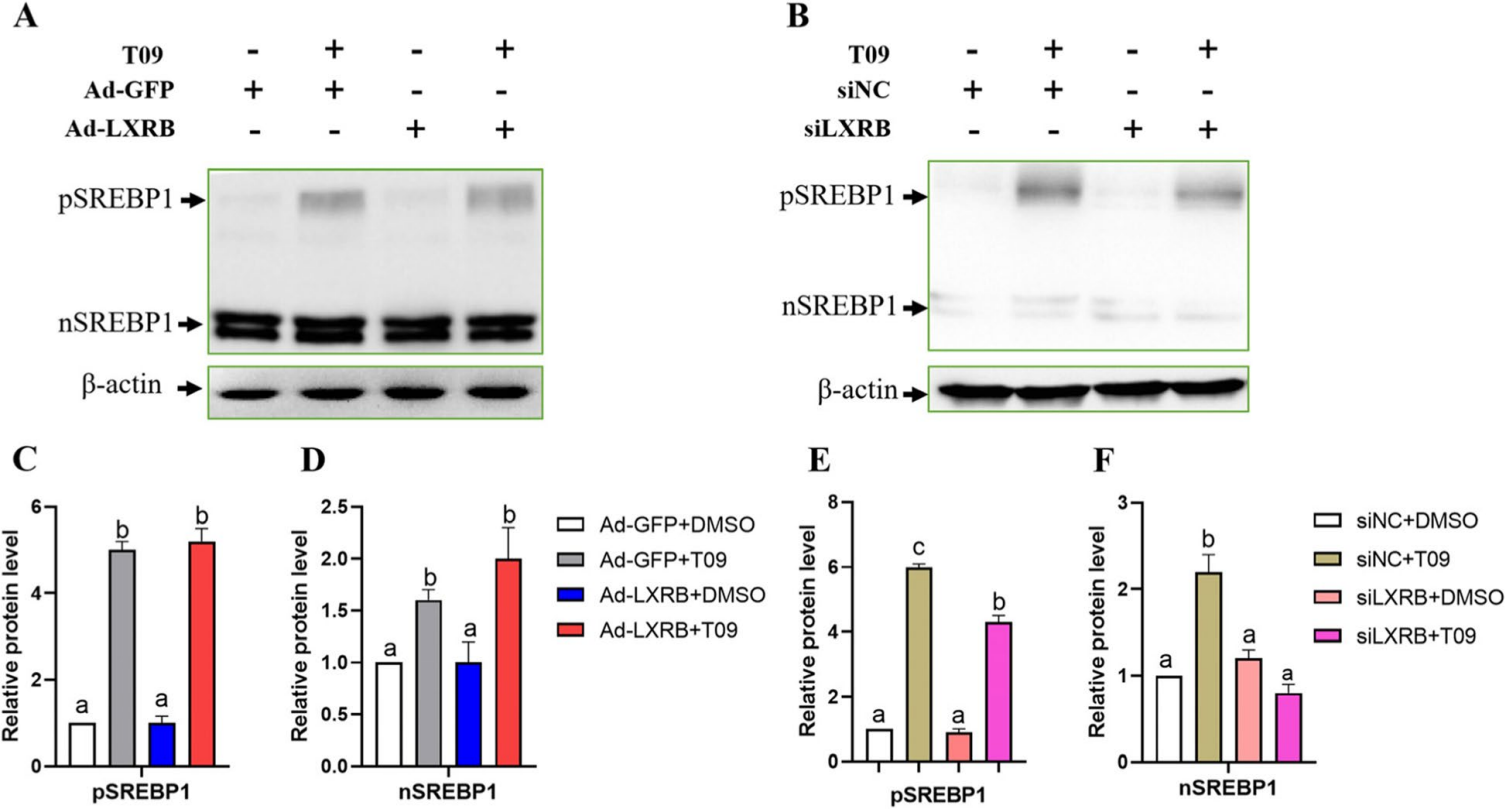
Overexpression or knockdown of LXRB alter the protein abundance of SREBP1 precursor (pSREBP1) and nuclear SREBP1 (nSREBP1) in goat mammary epithelial cells. Panel **A**: Western blot after overexpression of LXRB. Panel **B**: Western blot after knockdown of LXRB. Panel **C** and **E**: Quantitation of pSREBP1 level by Western blot. Panel **D** and **F**: Quantitation of nSREBP1 level by Western blot. Treatments were replicated 3 times. The Values are means ± SEM. The different letters denote significant (*P* < 0.05) differences

Compared with the control (Ad-GFP + DMSO), LXR activated by T09 significantly upregulated the abundance of pSREBP (*P* < 0.01) and nSREBP1(*P* = 0.01) (Fig. 7 A, C and D, *n* = 3). The GMEC incubated with Ad-LXRB or siLXRB alone had no significant effect (*P* > 0.05) on the protein level of pSREBP and nSREBP1. In the cells incubated with T09, knockdown of LXRB significantly downregulated the abundance of pSREBP (*P* = 0.04) and nSREBP1(*P* < 0.01), respectively (Fig. 7 B, E and F, *n* = 3).

**Fig. 8:**
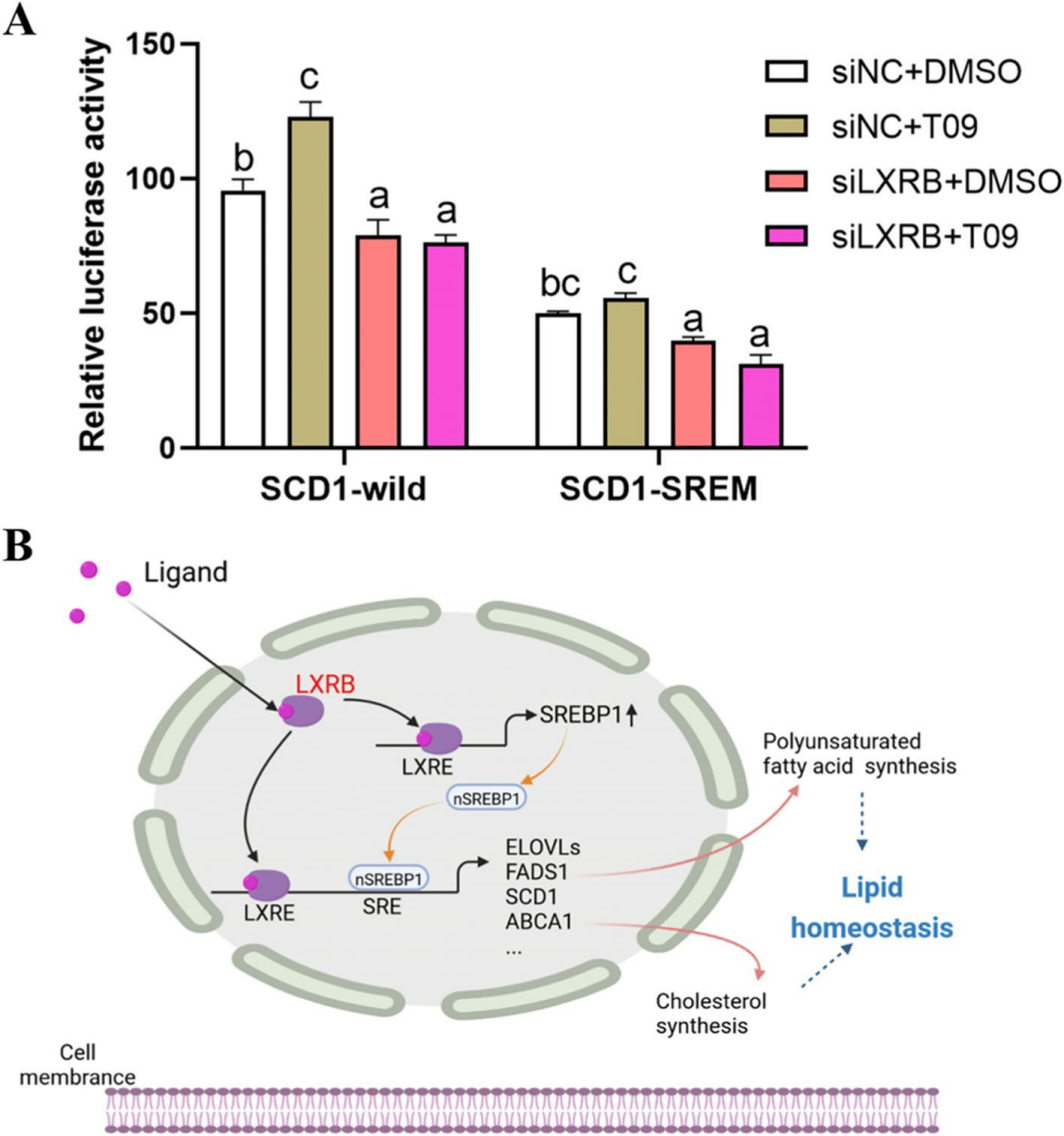
Knockdown of LXRB decreases the activity of SCD promoter and the schematic model of LXR-SREBP1 network regulating the cellular homeostasis. Panel **A**: The goat mammary epithelial cells were transfected with the activity of SCD1 promoter containing a wild type construct (SCD1-wild) or a mutation of the SREBP1 response element (SCD1-SREM) and incubated with the LXR agonist T09 or dimethyl sulfoxide (DMSO). Treatments were replicated 5 times. The values are means ± SEM. The different letters denote significant (*P* < 0.05) differences. Panel **B**: Model of LXRB-SREBP1 network regulating lipogenic homeostasis in goat mammary epithelial cells. After activated by the ligand, LXRB regulates several downstream lipogenic genes directly or in an SREBP1-dependent manner. The LXRB and SREBP1 network increased cellular polyunsaturated fatty acids through controlling the activity of stearoyl-CoA desaturase 1 (SCD1) and elongation of very long chain fatty acids protein 5, 6 and 7 (ELOVL5–7). These endogenous PUFA would act as natural ligands participating in milk synthesis and secretion and contribute the cellular homeostasis

The SCD1 promoter activity was assessed after knockdown of LXRB. As shown in Fig. 8A (*n* = 5), T09 treatment increased the activity of SCD1-wild (*P* = 0.02) compared with control (cells treated with DMSO) while its activity was also reduced by siLXRB. However, knockdown of LXRB blocked the induction of T09 on SCD1-wild activity (*P* > 0.05). T09 treatment had no significant effect on SCD1-SREM activity (*P* > 0.05) in the cell incubated with siNC while a lower activity of SCD1-SREM (*P* = 0.03) was observed in the cells incubated siLXRB compared with control (siNC + DMSO).

## Discussion

Although previous evidence suggested that the FADS enzyme family, SCD1 and ELOVLs are associated with the content of milk PUFA [41–43], the mechanism whereby synthesis of PUFA is controlled is unknown. In humans and rodents, LXR is a transcription factor regulating the desaturation and elongation of endogenous PUFA [31, 44]. A role of LXR in regulating the expression of desaturases (SCD1) was also reported in dairy goats [11] and cow [45, 46]. As the predominant subtype of LXR in ruminant mammary gland [32], however, whether LXRB plays a role in controlling the synthesis of PUFA in ruminant mammary cells has not been addressed. The present study is novel in that we studied the genes associated with PUFA synthesis and FA profiles through specific overexpressing or knockdown of LXRB protein abundance. Our data underscored an important role for LXRB in PUFA synthesis via controlling the processes of elongation and desaturation.

The ELOVL family is essential for FA elongation and synthesis of key saturated and PUFA in humans and rodents [42, 47]. In the current study, the higher levels of ELOVL5–7 in the cells incubated with T09 when LXRB was overexpressed and the lower levels of ELOVL5–7 when LXRB was knockdown suggested that they are targets of LXRB. The altered expression of ELOVL5 and ELOVL6 in the current study agreed with the data in the LXRB null mouse [30]. Together with the fact that the gene promoters of mouse ELOVL5 and goat ELOVL6 contain an LXRE site [14, 25], the pattern of expression for ELOVL5–6 with LXRB further confirmed that they are the direct targets of LXRB in the ruminant mammary gland. In the physiological context of ELOVLs and its products in the insulin sensitive mouse model [4, 48], our data also suggested a central role of LXRB in cellular homeostasis via regulating broad aspects of lipogenesis (Fig. 8 B). This idea agrees with the observation in mouse liver that the binding of LXRs to hepatic genes has broad effects on the transcriptional landscape beyond its canonical function as an activator of lipid metabolism genes [20].

Consistent with the pattern expression of ELOVL5–7 after overexpressing or knockdown of LXRB, the increased elongation index of C16 induced by T09 agrees with the findings in goats that ELOVL6 plays a role in the elongation of long-chain SFA (C16:0 to C18:0) while ELOVL5 and ELOVL7 are involved in the elongation of UFA containing 16 and 18 carbons [5, 7, 8]. In the cells containing T09, compared with the control, an increase of C18:2 after LXRB overexpression and a decrease of C18:2 after LXRB knockdown underscores a role of LXRB in enhancing the synthesis of PUFA. This finding agrees with data in goats [49] and humans [31]. Although evidence in macrophages suggested an increase of C20:4 and C20:5 upon activation of LXR [31], minor changes were observed for these PUFA in the present study. This response was likely due to the specific tissue differences for LXR activation. This idea is supported by the different roles of LXR subtypes on the regulation of SREBP1 expression in macrophages and mammary cells [31, 32]. The lack of goat-specific antibodies for ELOVLs  is clearly a limiting factor in the present study. Despite being unable to measure the protein level of ELOVL5–7, the present data illustrated that LXRB activation increased cellular PUFA (at least, C18:2) through controlling the activity of ELOVL5–7 (Fig. 8 B).

The desaturases work in concert with elongases during the endogenous biosynthesis of PUFA and in mouse are regulated by the activation of LXR [18]. The significant upregulation of *SCD1* level and its promoter activity after LXRB overexpression or activation by T09 agree with previous data in goats [10]. The upregulation of *SCD1* mRNA in the present study is consistent with the increased concentration of C16:1 and C18:1n9 when LXRB was activated. Along with the lack of alteration of SCD1 after knockdown of LXRA [10], the lower promoter activity of SCD1 in cells incubated with LXRB supports a predominant role of this subtype in regulating FA desaturation.

The FADS family catalyzes desaturation reactions at positions 5 and 6 of the fatty acyl chain during PUFA synthesis. The finding that a significant change for *FADS1* and a minor change for *FADS2* were observed in the current study suggests a specific regulatory role of LXRB in *FADS1* to control PUFA synthesis. The lack of effect of LXRB on *FADS2* in the present study agreed with the lack of change in concentrations of 22-carbon FA. This idea is supported by previous work in mouse [50]. Combined with the fact that the products of SCD1 or FADS1 can serve to activate various signaling pathways [51], the alterations in FA composition in the current study suggest that LXRB plays a role in the cellular homeostasis via controlling cellular synthesis of PUFA (Fig. 8 B). The idea is supported by the fact that repression of LXRs restricts macrophage biosynthesis of insulin-sensitizing omega-3 FA [50].

The accumulation of C16:0 could result in endoplasmic reticulum stress and apoptosis [6]. In the current study, the activation of LXRB in the mammary cells promoting the elongation and desaturation of C16:0 and C18:0 might serve to protect mammary cells from lipotoxicity. In ruminants, the *DGAT2* coding enzyme is involved in synthesis of TAG and is upregulated during lactation [35]. The upregulation of *PLIN2* and *DGAT2* when LXRB was overexpressed agreed with the accumulation of TAG in the present study. In the physiological context of PLIN2 for the formation and secretion of lipid droplets [52], the inducible increase of *PLIN2* in the present study suggested that LXRB activation would facilitate milk secretion. This idea was supported by the accumulation of TAG when LXRB was expressed.

ABCA1 is important in eliminating excess cholesterol from cells, and thus contributes to cellular homeostasis [53]. ABCA1 is induced by LXR in mouse liver [28]. The markedly high expression of ABCA1 in the cells incubated with T09 suggested it is a target gene of LXR in the goat mammary gland. The lower level of cholesterol in the cells incubated with T09 agreed with the role of ABCA1 in eliminating excess cholesterol [53]. It is worth noting that overexpression of LXRB upon T09 increased the level of cholesterol, suggesting a role of LXRB promoting cholesterol synthesis in the ruminant mammary gland (Fig. 8 B). This agreed with data in mouse liver [21, 27]. Because T09 activates both LXR subtypes, the modest change when LXRB was knockdown could have been due to LXRA eliciting a compensatory effect when LXRB expression is reduced. This idea is supported by data demonstrating that LXRA is prone to activate cholesterol metabolism [27]. The data in the current study suggested that LXRB is a key transcription factor that controls lipogenic homeostasis in the mammary gland.

Evidence from the promoter analysis suggested that LXR regulates several downstream lipogenic genes directly or in an SREBP1-dependent manner [11, 14, 32]. In the current study, the increases of SREBP1 and nSREBP1 protein abundance in the cells treated with T09 are consistent with the upregulation of *SREBP1* mRNA level [32]. Combined with the fact that the promoter of SREBP1 is mainly responsive to LXRB activation [32], the observation in cells containing T09 that LXRB knockdown decreased the abundance of SREBP1 and nSREBP1 suggests a direct mechanism of LXRB on SREBP1 at a transcriptional level.

To further assess the mechanism whereby LXRB controls its downstream genes, the *SCD1* promoter was used as a model in the current study. The lower activity of SCD1-LXRE-mut supported a crucial role for SREBP1 in regulating *SCD1* in the goat [11]. Along with the observation that activation of LXR by T09 had no significant effect for the SCD-SREM, the current data suggested that the full function of LXR in regulating lipogenesis is in an SREBP1-dependent manner in the goat mammary gland. Along with the finding that LXRA interference had a weak effect on *SCD1* promoter activity [10], the observation that knockdown of LXRB significantly decreased the level of *SCD1* promoter activity regardless of T09 treatment further underscores the important role LXRB in regulating lipogenesis in the ruminant mammary gland. Because dairy goats and dairy cows share conserved sequences for the lipogenic genes, the findings in the current study could be extrapolated to dairy cows. However, more comparative experiments across ruminant species would be required in the future. Collectively, the data in the current study highlighted an important role of the LXRB-SREBP1 network in PUFA synthesis through the genes encoding elongases and desaturases, and, thus, regulating lipid homeostasis in the goat mammary gland.

## Conclusion

The increase of PUFA content and genes encoding elongases and desaturases by LXRB activation demonstrated its important role in the synthesis of PUFA. The full lipogenic response elicited by LXRB is in an SREBP1-dependent fashion. In addition, the LXRB-SREBP1 network has a broad effect on cellular homeostasis via altering lipid droplet formation and cholesterol efflux. Although the present study cannot explain the full mechanisms of LXRB-SREBP1 network action in the lactating mammary gland, it seems closely related to the synthesis of PUFA. These endogenous PUFA would act as natural ligands participating in milk synthesis and secretion. Thus, controlling the activation of LXRB-SREBP1 network in the lactating mammary gland may provide possible strategies to improve the quality and production of milk.

## Supplementary Information

**Additional file 1:** **Table S1.** Name, accession number, sequences, amplicon length of primer pairs used in the present experiment, efficiency of amplification of PCR, and references. **Fig. S1.** Relative mRNA expression level in the goat mammary epithelial cells incubated with adenovirus expression liver X receptor beta (LXRB). **Fig. S2.** Relative mRNA expression level in the goat mammary epithelial cells incubated siRNA targeted liver X receptor beta (LXRB).

## Data Availability

All data measured or analyzed during this work are available from the corresponding author upon reasonable request.

## Abbreviations

ABCA1: ATP binding cassette subfamily a member 1
Ad-LXRB: Recombinant adenovirus expressing LXRB protein with a FLAG tag epitope
Ad-GFP: Recombinant adenovirus expressing green fluorescent protein
DGAT2: Diacylglycerol O-acyltransferase 2
LXRA: Liver X receptor alfa
LXRB: Liver X receptor beta
PLIN2: Adipose differentiation-related protein
FA: Fatty acid
ELOVL5: Elongation of very long chain fatty acids protein 5
ELOVL6: Elongation of very long chain fatty acids protein 6
ELOVL7: Elongation of very long chain fatty acids protein 7
FADS1: Fatty acid desaturase 1
FADS2: Fatty acid desaturase 2
GMEC: Goat mammary epithelial cells
pRL-TK: Renilla vector
PUFA: Polyunsaturated fatty acids
qPCR: Quantitative real-time PCR
SCD1: Stearoyl-CoA desaturase 1
SCD1-wild: SCD1 promoter
SCD1-SRM: SCD1 promoter with SREBP1 response element mutation
siLXRB: Small interference RNA targeted LXRB
siNC: Negative control for the small interference RNA
SREBP1: Sterol regulatory element binding transcription factor 1
UXT: Ubiquitously expressed transcript
TAG: Triacylglycerol
