# Supplementary material for: The LXRB-SREBP1 network regulates lipogenic homeostasis by controlling the synthesis of polyunsaturated fatty acids in goat mammary epithelial cells
Source: J Anim Sci Biotechnol. 2022 Nov 7;13:120. doi: 10.1186/s40104-022-00774-4 (PMC9639257; doi:10.1186/s40104-022-00774-4)
Supplement: Supplementary file 1 — Additional file 1: Table S1. Name, accession number, sequences, amplicon length of primer pairs used in the present experiment, efficiency of amplification of PCR, and references. Fig. S1. Relative mRNA expression level in the goat mammary epithelial cells incubated with adenovirus expression liver X receptor beta (LXRB). Fig. S2. Relative mRNA expression level in the goat mammary epithelial cells incubated siRNA targeted liver X receptor beta (LXRB). [file 40104_2022_774_MOESM1_ESM.docx]

**Table S1** Name, accession number, sequences, amplicon length of primer pairs used in the present experiment, efficiency of amplification of PCR, and references

| **Gene/Acc. #** | **Primers^1^** | **Sequence (5' to 3')** | **bp^2^** | **Efficiency^3^** | **Reference.** |
| --- | --- | --- | --- | --- | --- |
| *ABCA1* | F. 972 | CGGCGGCTTCTCTTGTATAGC | 101 | 2.16 | Bionaz and Loor [35] |
| DQ059505 | R.1072 | TTCAAGCGTGAGCTGAAACG |  |  |  |
| *DGAT2*  *HM566448.1* | F. 192  R. 291 | CATGTACACATTCTGCACCGATT  TGACCTCCTGCCACCTTTCT | 100 | 2.10 | Bionaz and Loor [35] |
| *FADS1*  *EE347846* | F. 552  R. 652 | GGTGGACTTGGCCTGGATG  TGACCATGAAGACAAGCCCC | 101 | 2.18 | Bionaz and Loor [35] |
| *FADS2*  *DV895683* | F. 192  R. 291 | AAAGGGTGCCTCTGCCAACT  ACACGTGCAGCATGTTCACA | 101 | 2.06 | Bionaz and Loor [35] |
| *ELOVL5* | F. 318 | catgaagatcatccgtgtgc | 198 | 1.97 | Shi et al. [5] |
| *NM_001285628.1* | R.515 | aagtgtggcgccaaagtaag |  |  |  |
| *ELOVL6* | F. 173 | GGAAGCCTTTAGTGCTCTGGTC | 205 | 1.94 | Shi et al. [7] |
| *NM_001102155.1* | R.377 | ATTGTATCTCCTAGTTCGGGTGC |  |  |  |
| *ELOVL7* | F. 308 | ACTATTCACAGTCGCCTACGG | 166 | 2.07 | Shi et al. [8] |
| *XM_005694673.2* | R.473 | CAGGTCCATGGCATGATGGT |  |  |  |
| *PLIN2* | F. 83 | TGGTCTCCTCGGCTTACATC | 268 | 2.07 | Shi et al. [52] |
| *NM_173980* | R.350 | TCTTTTGCCCCAGTCATAGC |  |  |  |
| *LXRB* | F.132 | CAGCCTCCGTTGTGGTCAT | 150 | 2.01 | This manuscript |
| *XM_012190735.2* | R.262 | CTTCACAGCTGAGCACGTTG |  |  |  |
| *SCD1* | F. 357 | CCATCGCCTGTGGAGTCAC | 256 | 1.92 | Shi et al. [38] |
| *GU947654* | R.612 | GTCGGATAAATCTAGCGTAGCA |  |  |  |
| *UXT* | F. 270 | TGTGGCCCTTGGATATGGTT | 101 | 2.06 | Bionaz and Loor [35] |
| *XM_005700842.1* | R.370 | GGTTGTCGCTGAGCTCTGTG |  |  |  |

^1^Primer direction (F-forward, R-reverse) and hybridization position on the sequence.

^2^Amplicon size in base pair (bp)

^3^Efficiency of amplification

**Fig. S1** Relative mRNA expression level in the goat mammary epithelial cells incubated with adenovirus expression liver X receptor beta (LXRB). For adenoviral infection, the GMEC cultured in six-well plates were incubated with adenovirus medium Ad-GFP or Ad-LXRB, respectively. Treated GMEC were cultured with 1 μmol/L T09 (diluted in dimethyl sulfoxide, DMSO at final concentration 0.1%, Sigma, St. Louis, MO, USA) or control (final DMSO at 0.1%, Sigma) after 24 h of initial culture, and then harvested at 48 h (24 h later) for RNA extraction

**Fig. S2** Relative mRNA expression level in the goat mammary epithelial cells incubated siRNA targeted liver X receptor beta (LXRB). For RNA interference, the GMEC cultured in six-well plates were transfected with siLXRB or control (siNC), respectively, using Lipofectamine^®^ RNAiMAX according to the manufacturer’s protocol (Invitrogen, USA). Treated GMEC were cultured with 1 μmol/L T09 (diluted in dimethyl sulfoxide, DMSO at final concentration 0.1%, Sigma, St. Louis, MO, USA) or control (final DMSO at 0.1%, Sigma) after 24 h of initial culture, and then harvested at 48 h (24 h later) for RNA extraction
